# Oral Presentations 5

**DOI:** 10.1038/sj.bjc.6601932

**Published:** 2004-06-22


### ORAL PRESENTATIONS 5

### 5.1

### A ROLE FOR A20 IN TUMOUR ANGIOGENESIS

HW Chng [1], R Camplejohn [1], I Hart [2], L Nicholson [1]
[1] King's College London, London, United Kingdom, [2] Tumour Biology Laboratory, Cancer Research UK, London, United Kingdom

The purpose of this study was to determine the role of A20 in angiogenic development. A20 was identified first as an immediate-early, tumour necrosis factor (TNF)-responsive gene with an unique zinc finger motif. Being an anti-apoptotic molecule, A20 has been shown to inhibit TNF-induced programmed death of human breast carcinoma cells (MCF 7), murine fibrosarcoma cells (WEHI 164), murine embryonic fibroblasts (NIH 3T3) and human umbilical vein endothelial cells (HUVECs). A20 also can inhibit apoptosis induced by other stimuli; including serum depletion of Louckes B cells, BJAB B cells and HUVECs, p53 expression in H1299 epithelial cells, and LPS treatment of human microvascular endothelial HMEC-1 cells. Our results from an *in situ* hybridisation assay on breast cancer tissue sections showed that expression of A20 was localised mainly to endothelium of tumour-associated blood vessels. These preliminary data suggested a novel role for A20 in cancer angiogenesis. Accordingly we decided to use a small RNA interference assay (sRNAi) to downregulate the expression of A20 in HUVECs and assess how this downregulated expression of A20 might influence tubule formation using an *in vitro* angiogenesis assay. We have confirmed that downregulated expression of A20 in HUVECs was achieved at both the protein and RNA levels by Western blot analysis (50% protein [as determined by densitometry] for A20 RNAi cells versus 100% for scrambled RNAi treated at 48 hours time-point) and quantitative Real-Time PCR (transcript levels reduced by 50% after RNAi treatment) respectively. More recently, in three independent experiments, we have shown this downregulated expression of A20 reduces the number of tubules (3.3 versus 4.0, $p \leq 0.01$) and tubule length (1.4 versus 1.6, $p \leq 0.01$) in the *in vitro* angiogenesis kit. These initial results appear to indicate an exciting and important new role for A20 in promoting tumour angiogenesis, possibly through mediating improved survival of endothelial cells.

### 5.2

### THERAPEUTIC EVALUATION OF THE VEGF RECEPTOR TYROSINE KINASE INHIBITOR ZD6474 IN A MURINE MODEL OF SPONTANEOUS COLORECTAL CANCER

RW Wilkinson [1], AJ Ryan [1], SR Wedge [1], ITG Pyrah [2], N Mandir [2], RA Goodlad [2]
[1] AstraZeneca, Macclesfield, United Kingdom, [2] Cancer Research UK, London, United Kingdom

ZD6474 is an orally active inhibitor of vascular endothelial growth factor receptor-2 (VEGFR-2, KDR) tyrosine kinase activity, with additional activity against epidermal growth factor receptor (EGFR) tyrosine kinase. This study set out to examine the therapeutic effect of ZD6474 on early colorectal tumourigenesis in multiple intestinal neoplasia (*Min*) mice. These mice spontaneously develop numerous benign polyps due to a mutation of the adenomatous polyposis coli (APC) gene, as in familial APC in humans. ZD6474 (12.5, 25 or 50 mg/kg/day, p.o.) or vehicle was administered to 6-week old *Min* mice (n=12) for 28 days. At autopsy, the small intestines and colons were isolated, rinsed, weighed, dissected longitudinally, spread onto filter paper, fixed in Carnoy's fixative for 3 hours and then stored in 70% ethanol. They were later assessed under a stereomicroscope for polyp number and diameter. Tumour burden was calculated as the product of polyp number and polyp volume. Additional satellite groups of mice (n=4) were taken for histological examination and immunohistochemistry (IHC).
In the small bowel, ZD6474 (50 mg/kg/day) was associated with a significant reduction both in mean polyp number per mouse (90.9 ± 20.7 vs 49.5 ± 12.9 in control vs treated, respectively, *P*=0.033) and in mean polyp diameter (1.18 ± 0.04 mm vs 0.95 ± 0.05 mm, *P*=0.005), resulting in a 75% decrease in polyp burden (76.5 ± 11.7 $mm^3$ vs 18.3 ± 5.6 $mm^3$, *P*=0.005). In addition, ZD6474 (50 mg/kg/day) treatment also resulted in fewer polyps in the colon (mean polyp number 3.46 ± 0.98 vs 0.83 ± 0.34 in control and treated, respectively, *P*=0.019).
This activity is likely to be a consequence of inhibiting VEGF/VEGFR-2 signalling during the early stages of tumour initiation and growth. Immunostaining with an antibody for von Willebrand Factor (vWF) to label vasculature, revealed strong staining in large polyps near the luminal surface. ZD6474 significantly reduces polyp growth and number in the *Min* mouse model of intestinal adenoma, and provides a scientific rationale for studying the effects of VEGFR-2 signalling inhibitors in early disease in the clinic. ZD6474 is currently in Phase II clinical development.

### 5.3

### ACUTE ANTI-VASCULAR EFFECTS OF COMBRETASTATIN A4 PHOSPHATE (CA-4-P) ON THE SW1222 TUMOUR AS MEASURED BY DYNAMIC CONTRAST ENHANCED (DCE)-MRI

KJ Lankester [1], RJ Maxwell [1], RB Pedley [2], SA Hill [1], GJS Rustin [3], GM Tozer [1]
[1] Gray Cancer Institute, Northwood, Middlesex, United Kingdom, [2] Dept of Oncology, Royal Free Hospital, London, United Kingdom, [3] Dept of Medical Oncology, Mount Vernon Hospital, Northwood, Middleesex, United Kingdom

CA-4-P (200mg/kg) plus radioimmunotherapy (with anti-CEA antibody, $^{131}$I-A5B7) can cure SW1222 tumours (human colon carcinoma xenograft) in mice. A phase I trial of this regimen has started with DCE-MRI to measure the acute anti-vascular effects of CA-4-P (using tissue uptake of contrast agent, Gd-DTPA). We used DCE-MRI to measure SW1222 response to CA-4-P at 30mg/kg (clinically relevant dose), 100mg/kg and 200mg/kg in order to aid trial results.
MF1 nude mice with SW1222 tumours implanted subcutaneously were treated with either saline (control) or 30, 100 or 200 mg/kg of CA-4-P i.p. then imaged 4 or 24 hours post-injection. Tumour IAUGC (initial area under the Gd-DTPA concentration-time curve) was measured. The effect of CA-4-P on $^{131}$I-A5B7 retention in tumour 96 hours after injection was also determined.
All doses caused reduction in tumour 4 hours after CA-4-P injection (38%, 71% and 86% decrease vs control for 30, 100 and 200mg/kg respectively, all $p<0.05$). At 24 hours, recovery was seen at 30mg/kg but reduction in IAUGC persisted at 100mg/kg (not significant) and at 200mg/kg (58% decrease, $p<0.05$). Correlation coefficients for dose-response were -0.88 ($p<0.0001$) and –0.59 ($p<0.01$) at 4 and 24 hours. Spatial heterogeneity was seen with a significant difference between tumour centre and rim in baseline IAUGC and in response to CA-4-P at 100 & 200mg/kg at 4 hours. The magnitude in reduction of IAUGC at 30 mg/kg was similar to that seen in patients with CA-4-P (dose ≥52mg/$m^2$). Antibody retention was significantly increased vs control for all dose levels (by 71%, 78%, and 90% for 30, 100 and 200mg/kg respectively). Results show that low (clinically relevant) doses of CA-4-P effectively enhance antibody retention despite only transient vascular effects.
Funding: Cancer Research UK, Marie Curie Translational Research Trust, Cancer Treatment and Research Trust.

### 5.4

### TUMOUR MICROVASCULATURE ASSESSMENT BY DYNAMIC CONTRAST-ENHANCED MRI FAILS TO CORRELATE WITH VEGF EXPRESSION IN BREAST CANCER BIOPSIES

MW Ah-See [1], AR Padhani [1], NJ Taylor [1], FM Daley [2], SM Bentzen [2], RJ Burcombe [1], M Harrison [1], JJ Stirling [1], MO Leach [3], A Makris [1]
[1] Mount Vernon Hospital, Northwood, United Kingdom, [2] Gray Cancer Institute, Northwood, United Kingdom, [3] Royal Marsden Hospital, Sutton, United Kingdom

**Background:** Vascular endothelial growth factor (VEGF) is the principal angiogenic factor driving neovascularisation within breast cancers. Multi-functional dynamic contrast-enhanced MRI (DCE-MRI) provides a method for assessing tumour microvasculature. Here we test the possible correlation between tumour vascularity parameters as assessed by DCE-MRI & VEGF expression in breast tumour biopsies.
**Materials and Methods:** 20 patients with biopsy-proven primary breast cancer (median age 44 years, range 29-58) were imaged prior to treatment. DCE-MRI was performed using gadolinium & parametric images were calculated reflecting microvessel permeability ($K^{trans}$, $v_e$, MaxGd), perfusion (rBV, rBF, MTT) & oxygenation ($R_2$*). Median values for each parameter were derived from whole tumour regions of interest. The expression of VEGF in the diagnostic biopsies was analysed by immunohistochemistry using the anti-VEGF monoclonal antibody JH121 (Neomarker). The intensity & percentage of VEGF staining was scored & a VEGF immunoreactive score was calculated as the product of the 2 factors. The association between 2 parameters was quantified by Spearman's rank correlation coefficient, $r_s$, & the statistical significance was the 2-tailed P-value for rejecting the null hypothesis.
**Results:** $K^{trans}$ & $R_2$* correlated significantly with the perfusion parameters rBF ($r_s$ =0.60, $p<0.01$ & $r_s$ =-0.68, $p<0.01$ respectively) & rBV ($r_s$ = 0.55, $p<0.05$ & $r_s$ =-0.67, $p<0.01$ respectively). The median VEGF immunoreactive score was 6 (range 1-12). No correlation was found between tumour VEGF expression & the pre-treatment vascular parameter values as assessed by DCE-MRI.
**Conclusions:** A strong correlation was demonstrated between the DCE-MRI-derived vascular parameters reflecting tumour microvessel permeability, perfusion and oxygenation. No significant correlation was seen between tumour VEGF expression & DCE-MRI-derived vascular parameters, which may be due to small patient number within the study or disparity between visible & functional tumour microvasculature.

© 2004 Cancer Research UK

### 5.5

### VEGF, VEGF RECEPTORS AND ANGIOGENESIS IN HAPATOCELLULAR CARCINOMA

Z Gao [1], YL Zhu [2], B Wang [2], DX Yu [2]
[1] Department of Medical Biology, Weifang Medical University, Weifang, China, [2] Department of Radiology, Weifang, China

**Introduction** To evaluate the expression of vascular endothelial growth factor (VEGF) and its receptors in the angiogenesis of HCC.
**Materials and Methods** Thirty-nine cases (41 lesions) of HCC were proved surgically. Clinicopathological characteristics were analysed. The expressions of VEGF, Flt-1 and KDR/Flk-1 were detected with immunohistochemical SP method. The clinical and histopathological characteristics of HCC were compared with the immunohistochemical results of VEGF, Flt-1 and KDR/Flk-1.
**Results** The diameter was equal to or smaller than 5 cm in 22 lesions (22/41, 53.7%), the diameter was larger than 5 cm in 19 lesions (19/41, 46.3%); pseudocapsula were found in 12 lesions (12/41, 29.3%), incomplete pseudocapsula or non-pseudocapsula were found in 29 lesions (29/41, 70.7%). There were 11 lesions (11/41, 26.8%) in high potential invasion and metastasis group (intrahepatic daughter foci, tumor-emboli in portal veins, lymphaden metastasis), the others were 30 lesions (30/41, 73.2%) in low potential invasion and metastasis group. 32 lesions (32/41, 78.0%) were accompanied by hepatic cirrhosis and the others (9/41, 22.0%) were not. Immunohistochemical results showed that the positive rate of VEGF, Flt-1 and KDR/Flk-1 expression was 61.0% (25/41), 68.3% (28/41) and 70.7% (29/41) respectively. VEGF and KDR/Flk-1 expression were significantly different between the group of high potential invasion and metastasis and low potential invasion and metastasis ($P < 0.05$), the group of HCC with incomplete pseudocapsula or without pseudocapsula and complete pseudocapsula ($P < 0.01$), and the group of lesions whose diameter equal to or smaller than 5 cm and larger than 5 cm ($P < 0.05$). It showed that VEGF expression correlated with KDR/Flk-1 ($P < 0.001$). No correlations were found between Flt-1 and VEGF or KDR/Flk-1.
**Conclusions** These results suggest that VEGF and KDR/Flk-1 play a potential important role in the angiogenesis, growth and metastasis of HCC.

### 5.6

### TARGETING THE VASCULATURE – A NEW THERAPUETIC STRATEGY FOR DELAYING THE GROWTH OF EWING'S SARCOMA FAMILY OF TUMOURS (ESFT)

S Dalal [1], C Cullinane [2], S.A. Burchill [1]
[1] United Kingdom, Cancer Research UK Clinical Centre, St. James's University Hospital, Leeds, [2] United Kingdom, Department of Pathology, St. James's University Hospital, Leeds

Vascular endothelial growth factor (VEGF) and placental growth factor (PlGF) are the key regulators of angiogenesis in ESFT (Dalal *et al*., 2003, AACR, 44:3047), thus making them attractive targets for new therapeutic strategies in this tumour group. In this study the effects of anti-VEGF compounds rhuMAb-VEGF (Bevacizumab) and VEGF Trap, anti-receptor tyrosine kinase compounds SU6668 and SU5416 and anti-vascular agents combretastatin CA4, CA4 phosphate prodrug (CA4P) and ZD6126 on the growth of subcutaneous (sc) ESFT in mice have been studied. Mice were injected in a single sc site on the flank with ESFT RD-ES cells (5x$10^6$). On day 15, mice were injected ip/sc with rhuMAb-VEGF (10mg/kg/x2 or x4/week; n=7-8), VEGF Trap (2.5 or 25mg/kg/x2/wk; n=5), SU6668 (100mg/kg/day; n=10), SU5416 (25mg/kg/day; n=10), CA4/CA4P (50mg/kg/day; n=7) or ZD6126 (100mg/kg/day; n=8). Tumour size was measured twice weekly. Once tumours reached 1.4$cm^2$ or at a fixed time point, mice were sacrificed, tumours excised, fixed and embedded in paraffin. Histology of tumours was examined by light microscopy after staining sections with H&E. Treatment with rhuMAb-VEGF, VEGF Trap, SU6668 and SU5416 significantly delayed tumour growth (p<0.001 for all groups), the efficacy of which was not dependent on initial tumour size. However, x4/week rhuMAb-VEGF was not more effective than x2/week rhuMAb-VEGF. Histology of treated tumours revealed geographical necrosis and reduced vascularisation compared to untreated groups. Initial tumour growth was also delayed following treatment with CA4, CA4P and ZD6126, with these agents having a striking effect on tumour histology. All treated tumours displayed large central areas of necrosis with no viable cells, however a peripheral rim of viable proliferating tumour cells surrounded this.
Further studies are underway to fully investigate the activity of these compounds in ESFT.

### 5.7

### EFFECTS OF VARIOUS HEPARIN FRACTIONS OF DIFFERING MOLECULAR COMPOSITION ON PRIMARY TUMOUR GROWTH, ANGIOGENESIS AND APOPTOSIS IN A HEPARIN DEFICIENT TRANSGENIC MOUSE MODEL

F A Nasir [1], H K Patel [2], A K Kakkar [1]
[1] Department of Surgical Oncology and Technology, Imperial College, London, United Kingdom, [2] Thrombosis Research Institute, London, United Kingdom

Low molecular weight heparin therapy (LMWH) prolongs survival in patients with solid tumour malignancy. The mechanism in unclear but is independent of the prevention of fatal thromboembolic events. We have studied the effects of various heparin fractions of differing molecular composition on primary tumour growth, angiogenesis and apoptosis in a heparin deficient transgenic mouse model.
Mice deficient by gene knockout, for the enzyme N-deacytylase/N-sulphotransferase (NDST-2) and unable to synthesize cellular heparin, were injected subcutaneously in the flank with 2x$10^6$ B16/F10 murine melanoma cells, and randomly allocated to receive N/saline (n=36) Unfractionated heparin UFH (150 ug) (n=21), LMWH dalteparin(50 ug) (n-23) or Pentasaccharide (PS)(10ug) (n=21). Tumour volumes were measured daily to day 15 to assess tumour growth and paraffin embedded sections of tumours was evaluated for apoptosis (TUNEL assay) and angiogenesis (after immunostaining with Anti-CD 31 to count microvessels per high powered field). Differences among groups were analysed using a one-way analysis of variance and individual comparison against using control using the Dunnet test.

| | Control | uFH | PS | LMWH |
|---|---|---|---|---|
| Tumour Vol/ mean ± SE<br>P vs control | 108 ± 9 | 84 ± 11<br>n.s | 72 ± 12<br>0.03 | 61 ± 9<br>0.002 |
| Microvessel density/ hpf<br>P vs control | 24 ± 2.9 | 20 ± 2.9<br>n.s | 13 ± 3<br>0.02 | 12 ± 2.6<br>0.008 |
| Apoptotic index (%)<br>P vs control | 3.8 ± 03 | 4.7 ± 0.6<br>n.s | 6.4 ± 0.5<br>n.s | 11.3 ± 1.5<br>0.0001 |

These data indicate that the LMWH dalteparin is able to inhibit tumour growth through inhibition of angiogenesis and induction of apoptosis, and suggest a mechanism requiring more than just the inhibition of blood coagulation activation, through the inhibition of factor Xa, for its ability to induce these two powerful anti-tumour mechanisms.

### 5.8

### DIFFERENTIATION MODULATES THE HYPOXIA-INDUCIBLE GENE PROGRAM IN MACROPHAGES: EFFECTS OF ALTERATIONS IN IRON HOMEOSTASIS

HJ Knowles [1], PJ Ratcliffe [2], AL Harris [1]
[1] Cancer Research UK, Molecular Oncology Laboratory, Weatherall Institute of Molecular Medicine, John Radcliffe Hospital, Oxford, United Kingdom, [2] The Henry Wellcome Building of Genomic Medicine, University of Oxford, Roosevelt Drive, Oxford, United Kingdom

Macrophage infiltration into solid tumours is associated with poor prognosis, tumour angiogenesis and high levels of VEGF. Hypoxia attracts tumour-associated macrophages (TAMs) into avascular areas, where they express the hypoxia-inducible transcription factors HIF-1α and HIF-2α. However, the additional presence of some HIF-positive TAMs in vascular (and presumably well oxygenated) areas suggests that the HIF pathway in macrophages may be regulated by factors other than hypoxia.
In vitro differentiation of monocytic cell lines (THP1, U937) with 16 nM phorbol 12-myristate 13-acetate (PMA) caused upregulation of HIF-2α mRNA as detected by ribonuclease protection assay (fold induction 5.64 ± 3.4, $P < 0.01$) and induced expression of both HIF-1α and HIF-2α protein. Both proteins were further inducible by hypoxia (16 h 0.1% $O_2$). Presence of differentiation-induced HIF was not due to altered expression of the HIF-regulating PHD enzymes (PHD 1-3). However, this HIF was non-hydroxylated and both 25 µM ascorbate and 50 µM $FeCl_2$ antagonised its induction by PMA-differentiation under normoxia, suggesting that PHD enzyme activity is sub-maximal in differentiated cells. This may be due to the reduced total iron content in differentiated macrophages compared with monocytic cells (52.3% ± 27.4% iron as measured by ferrozine colorimetric assay, $P < 0.05$). We have also analysed changes in the intracellular labile iron pool using the fluorescent iron chelator calcein-AM. We have investigated the expression of iron-regulatory proteins during differentiation (e.g. transferrin receptor, Nramp1) and their association with induction of HIF-α.
These data point to a clear change in the regulation of hypoxia-inducible gene expression in macrophages, involving modulation of HIF and altered iron homeostasis. This may be an adaptation of macrophages to prolonged hypoxic exposure in solid tumour tissue.

